# A Review of Radiotherapy-Induced Late Effects Research after Advanced Technology Treatments

**DOI:** 10.3389/fonc.2016.00013

**Published:** 2016-02-10

**Authors:** Wayne D. Newhauser, Amy Berrington de Gonzalez, Reinhard Schulte, Choonsik Lee

**Affiliations:** ^1^Department of Physics and Astronomy, Louisiana State University, Baton Rouge, LA, USA; ^2^Department of Physics, Mary Bird Perkins Cancer Center, Baton Rouge, LA, USA; ^3^Radiation Epidemiology Branch, National Institutes of Health, Rockville, MD, USA; ^4^Department of Basic Sciences, Loma Linda University Medical Center, Loma Linda, CA, USA

**Keywords:** late effects, dose, risk, measurement, calculation, proton, photon

## Abstract

The number of incident cancers and long-term cancer survivors is expected to increase substantially for at least a decade. Advanced technology radiotherapies, e.g., using beams of protons and photons, offer dosimetric advantages that theoretically yield better outcomes. In general, evidence from controlled clinical trials and epidemiology studies are lacking. To conduct these studies, new research methods and infrastructure will be needed. In the paper, we review several key research methods of relevance to late effects after advanced technology proton-beam and photon-beam radiotherapies. In particular, we focus on the determination of exposures to therapeutic and stray radiation and related uncertainties, with discussion of recent advances in exposure calculation methods, uncertainties, *in silico* studies, computing infrastructure, electronic medical records, and risk visualization. We identify six key areas of methodology and infrastructure that will be needed to conduct future outcome studies of radiation late effects.

## Introduction

About one in two men and women born today will be diagnosed with some form of cancer in their lifetime (1). Worldwide cancer incidence in 2012 was estimated at 14.1 million new cases and 8.2 million deaths (2). In the United States, cancer incidence rates are projected to generally stabilize over the next decade, but the incidence will increase by more than 20% due to changes in demographics (3). Almost two-thirds of all cancer patients receive some form of radiation therapy during the course of treatment (4), predominantly with external-beam *photon therapy*. Treatments with beams of charged particles have become popular, especially *proton therapy* (5), and a few heavier charged particle facilities have been built in Europe and Asia for research and patient care. The main rationale for using charged particle beams is that they sterilize the tumor, like X-ray therapies, while delivering less radiation dose to healthy tissues (6, 7).

Advances in radiation therapy have contributed to improvements in long-term outcomes for cancer patients. For example, 5-year survival of cancer in the United States has increased to approximately 68% in adults and 83% in children (8). By 2020, there will be almost 20 million cancer survivors in the United States (9). Long-term survivors are at increased risk to develop treatment-induced side effects, such as radiogenic second cancer, complications of the cardiovascular (10, 11) and central nervous (12, 13) systems, fertility problems (14), and myriad other toxicities (15). These problems can be caused by disease (e.g., damage caused by a primary cancer) or by medical care, such as surgery, chemotherapy, and radiation therapy. For many patients, these will play out long after the primary cancer is cured. For example, in survivors of childhood cancer, the risks of morbidity and mortality remain elevated beyond the fourth decade of life (16).

The link between radiation therapy and several serious late effects has been well documented in the literature. Radiation epidemiology studies revealed increased risk for subsequent cancers after radiotherapy (17). One of the most striking examples being the cumulative risk of subsequent breast cancer after radiotherapy for Hodgkin’s lymphoma of 30% by age 55 (18). The clinical oncology literature reports that radiation is implicated in many subsequent cancers (19) and that mortality of primary cancers is decreasing, with increases in rates of mortality attributable to subsequent neoplasms, cardiac death, and pulmonary death largely due to treatment-related causes (20). Although radiotherapy significantly reduces breast cancer mortality and recurrence, the heart dose from older radiation treatments was found to materially impact total long-term survival (21). For some types of cancers, and in some pediatric cancers, second cancers can cause more deaths than the primary cancers (22). Second cancers account for 17–19% of all cancers, and, as a group, are one of the most common cancers in the USA (23). In adults, the proportion of second cancers related to radiotherapy was estimated at approximately 8% on average, with proportions varying from 4 to 24% for the specific sites considered (24). Corresponding estimates for children are not currently known but are likely to be considerably higher, given the increased radiosensitivity of children to some cancers and generally longer survival. For these and other reasons, the assessment of risks of late effects after radiotherapy has received increasing attention in the literature, including the impact of advanced technologies on outcomes (25, 26).

Advances in technology seek to improve cancer outcomes in two major ways, namely, by irradiating cancerous tissues in ways that lead to improved control of tumors and by reducing doses to healthy tissues to reduce treatment complications. Many advanced technologies have been implemented to further these goals, including treatment systems that use modulated beams of photons and protons. Unlike most new medical devices and drugs, advanced RTs are being widely deployed based on predicted improvements in outcome rather than superiority observed in prospective randomized clinical trials. The necessity of such trials is controversial (27). Furthermore, large economic forces are at play; proton and heavy-ion treatment units are the most expensive medical devices on the market and are perceived as a potentially disruptive technology in oncology. Clearly, additional new scientific approaches and knowledge would help to inform decision making in the future (28).

Currently, major gaps in scientific knowledge include (1) the long-term health problems of long-term cancer survivors, especially a decade or more after exposure; (2) the impact of radiation modality, dose, quality, and fractionation on the risk of late effects; (3) the applicability of risk models derived from healthy populations exposed to lower dose radiation to high-dose fractionated exposures in populations of cancer survivors; (4) the applicability of population-based risk models to individual patients, whose sensitivity to radiogenic late effects may vary with genetic profile and other factors; and (5) the impact of late effects after *advanced technology radiotherapies*, including incidence, severity, and economic considerations. Filling these gaps will require new research strategies, methods, and infrastructure.

The objective of this manuscript is to provide a review of selected research methodologies for radiogenic late effects after advanced technology radiation therapies. More specifically, we review aspects of relevance to proton-beam and photon-beam radiotherapies. We focus on craniospinal irradiation (CSI) as an illustrative example treatment to highlight current research capabilities and their limitations as this is one of the treatments for which proton therapy could be very beneficial. In particular, we review the determination of exposures to therapeutic and stray radiation and related uncertainties in the context of radiation late effects.

## Recent Advances in Research Methods

A few introductory remarks are necessary to provide a context for the thrust of our review. In the last decade, considerable progress has been made toward research methods of relevance to the risk of late effects after advanced technology radiation therapies. However, long-term clinical and epidemiological outcome studies of charged particle therapies are scarce (29). These studies are difficult to perform for many reasons, including a lack of dose and risk assessment tools that are suitable for prospective outcome studies. Currently, most photon therapy outcome studies are performed retrospectively, with the doses being reconstructed by specialists using proprietary research tools (30). Similar tools for charged particle are nascent.

To review progress and explore limitations of current tools, we shall consider the illustrative case of CSI for medulloblastoma. This is a particularly interesting case because it is common among pediatric brain tumors; approximately 80% of patients survive 5 years or more (31); the therapeutic radiation fields are large, variable in size, shape, and anatomic location (32); the therapeutic and stray radiation impacts many healthy tissues and organs (33); patients vary in age at diagnosis (34) and anatomic stature; it is commonly delivered with photon or proton beams; there are large differences in predicted doses and risks between photon and proton beams (35); genomics strongly influence outcomes (36); dose and risk assessments are technically challenging (26); and there is sufficient recent literature to form a coherent picture. The methodological literature includes dose measurements; dose calculations using clinical treatment planning systems (TPSs), *Monte Carlo simulations*, and *analytical models*; radiation quality; risk models; and other aspects.

Craniospinal irradiation attempts to limit the administration of tumor-sterilizing doses to several target volumes, including the spinal axis, the cranium, and a surgical resection volume near the posterior fossa. However, even the most advanced radiotherapies deliver low levels of stray radiation to the patient’s whole body. Observational data for various treatment sites revealed that nearly nine of every 10 subsequent tumors develop outside PTV (37). Thus, to fully understand late effects after external beam radiotherapy, one must, at a minimum, determine exposures from therapeutic and stray radiation to all of the organs and tissues of the body.

### Assessment of Radiation Exposure

Today, there are four major methods to determine radiation exposure from external beam radiotherapy, including measurements, analytical calculations (dose algorithms embedded in TPSs and nascent stand-alone algorithms), and Monte Carlo simulations.

Traditionally, measurements are used to develop, configure, and test dose calculation methods of various kinds, including pencil beam algorithms in clinical TPSs, research Monte Carlo codes, and other dose models. Methodologies for the measurement of therapeutic doses are generally well established and straightforward. Measurements of stray radiation are challenging and less well established. In particular, stray neutron radiation is experimentally difficult and subject to large uncertainties. High-energy photon beams produce neutrons via photoneutron interactions, and high-energy protons liberate neutrons via nuclear reactions.

**Figure 1:**
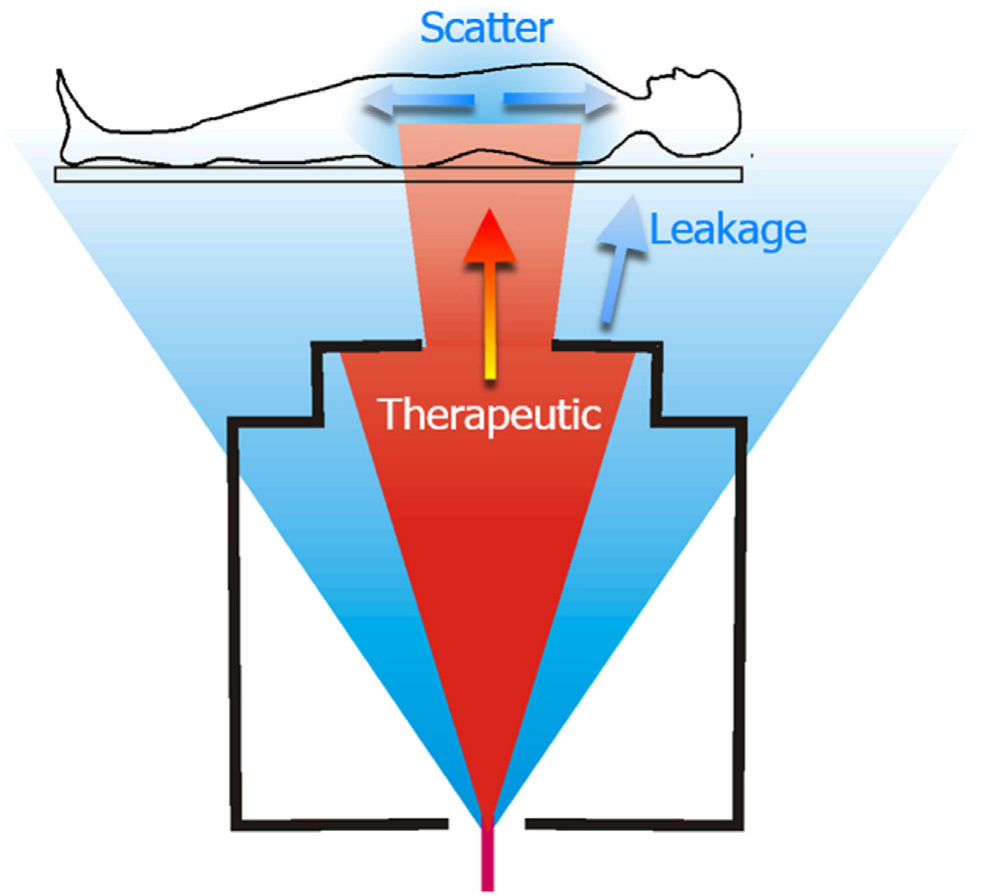
**Schematic diagram of a proton therapy treatment of the spinal axis**. The therapeutic dose is shown in red and unwanted stray radiation is shown in blue. The stray radiation comprises leakage radiation emanating from the treatment machine, and scatter radiation that is produced as the therapeutic radiation interacts with the patient. Figure from Ref. (26).

**Figure 2:**
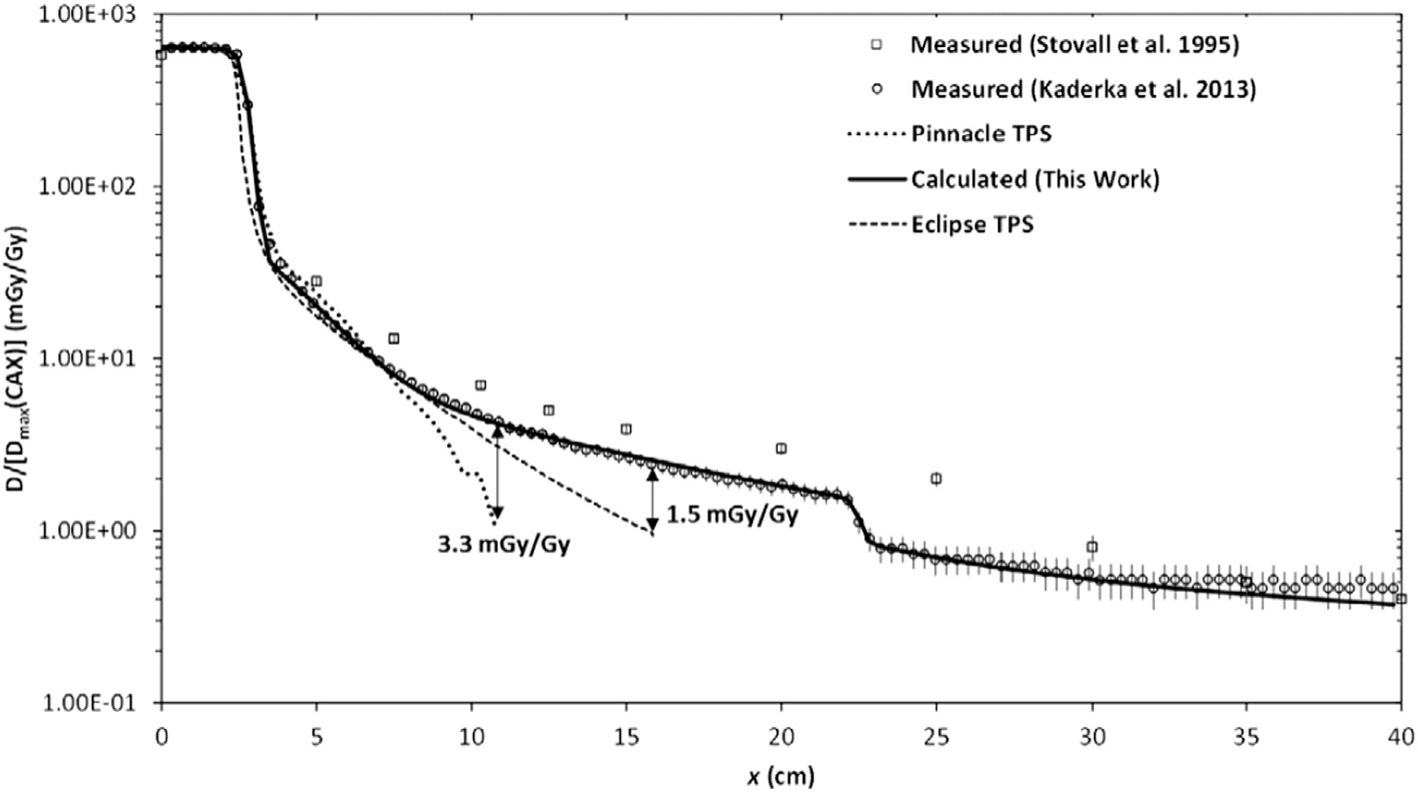
**Absorbed dose *D* as a function of lateral distance *x* from the photon therapy beam**. The calculated and measured absorbed dose values are normalized to the maximum therapeutic dose at the central axis *D*_max_(CAX). The radiation fields were produced by electron linear accelerator. Figure from Ref. (38).

Analytical dose algorithms are typically used in TPSs. They generally provide excellent dosimetric accuracy inside the therapeutic field and fast computation speeds. However, they severely underestimate stray radiation exposures, especially leakage radiation emanating from the treatment unit (38). Consequently, they are not suitable for research on the effects of radiation in that region (Figure 1). Recently, analytical models have been developed to calculate both therapeutic and stray radiotherapy exposures (Figure 2). Analytical models for whole-body exposure assessments are the least well developed of the four methods.

Monte Carlo simulations are generally well suited for research studies that require calculations of radiation exposures both inside and outside the treatment field. Of the four methods discussed here, the Monte Carlo method has advanced the most in recent years. For example, whole-body dose assessments that were computationally intractable in 2000 are now feasible in a research setting. This progress is attributable to refinements in general-purpose Monte Carlo codes, their adaptation to radiotherapy applications, and advances in parallel computing and low-cost electronic memory. Most Monte Carlo systems for radiotherapy simulations are built on general-purpose, full-featured codes, such as MCNP/X (39) and FLUKA (40, 41) with additional radiotherapy pre- and post-processing codes (42, 43), or with toolkits, such as GEANT4 (44) with radiotherapy packages (45). Important features include good interaction data and models, advanced source modeling and tallying features, parallel computing capability, variance reduction options, and statistical tests for convergence. Despite stunning breakthroughs in capabilities, today the Monte Carlo simulation method is seldom used for clinical treatment planning. One of the main reasons is that the method requires *high-performance computing* resources.

The selection of a dose assessment method involves consideration of the requirements of a particular study and the performance characteristics of available methods. Currently, no single method meets all the commonly encountered requirements on speed, accuracy, cost, and convenience. Consequently, most research studies require two or more methods to determine radiation doses. Traditionally, late-effect studies have utilized TPS calculations of therapeutic radiation dose and analytical calculations of stray radiation, where both methods were validated against measurements. In recent years, Monte Carlo simulations have played an increasing role. In the remainder of this manuscript, we focus on aspects of these methods that are of greatest relevance to researching the late effects from advanced technology radiotherapies, i.e., photon and proton beams. In the last part of this section, we mention the key advances in other disciplines that have had an enabling effect on the research methodologies presented here.

#### Proton Therapy

Interest in the late effects after proton radiation therapy has increased dramatically since the turn of the century, perhaps in part because of early publications on stray neutron exposures and because of the late toxicities observed after neutron beam therapy in previous decades. The latter experience tells a cautionary tale of the latent dangers of any new form of radiation treatment. In retrospect, it is remarkable that clinical proton therapy was practiced more than four decades before the first publications on stray neutron exposures appeared. Neutrons are of particular concern given their higher, but very uncertain, relative biological effectiveness (RBE) in humans. Neutron RBE values ware derived primarily from experimental data because, to date, there have not been any epidemiological studies that have been able to compare the risks with those of photon irradiation directly in a sufficient sample size. Binns and Hough (46) reported the first measurements of neutron exposures in developmental proton therapy beamline, which were alarmingly high. However, that beamline was never used for patient treatments because of the excessive neutron exposures. In 1998, Agosteo et al. published a seminal paper that reported on Monte Carlo simulations of stray photon and neutron exposures from proton therapy beamlines (47). Yan et al. (48) reported the first clinically relevant measurements of neutron spectra and exposures. They characterized each of the three heavily used clinical beamlines at the Harvard Cyclotron Laboratory (HCL) using multiple measurement techniques. The results of these three studies suggested that neutron exposures were not negligible and that careful attention should be paid to characterizing and minimizing neutron exposures for clinically used proton beamlines.

Indeed, a few years on, Hall (49) rescaled published neutron exposure data from the Harvard Cyclotron Laboratory (HCL) with simplistic risk calculations. He opined that passively scattered proton therapy may not be indicated for some patients, especially children, because of the second cancer risks from the whole-body neutron exposures. Although the key assumptions in his paper would be ultimately proven incorrect, the underlying concerns were justified. Specifically, a large international expansion of proton therapy had begun without due diligence regarding neutron exposures and their consequences.

By the time of Hall’s paper, systematic investigations of neutron exposures from proton therapy were already underway. Beginning in 2005, a series of reports was published that studies the systematics of therapeutic proton and stray neutron exposures, such as their dependence on proton-beam energy, field size, range modulation width, depth in phantom, collimator thickness, and other treatment factors (43, 50–60). The systematics were mostly investigated using general-purpose Monte Carlo simulations, a bellwether of the increasingly important role that Monte Carlo holds in radiotherapy research. Subsequent confirmatory measurements have been comparatively sparse but important; benchmark measurements confirmed the high-energy neutron physics models in Monte Carlo codes (61); end-to-end benchmarking confirmed Monte Carlo models of diverse clinically used proton beamlines (50, 62); and code intercomparisons further increased confidence in Monte Carlo simulations for clinically relevant beamlines, such as that in Ref. (63).

Recently, increased attention has been paid to developing reference data on neutron exposures (64), on methodology to experimentally benchmark predictive models (65), and to estimate mean radiation weighting factors and RBE values for neutrons (66–68).

By 2009, research methodologies had advanced sufficiently to allow for the first dose and theoretical risk assessment study, which was reported for CSI that included both therapeutic and stray neutron radiation (33). The modeling included a refined and more complete analysis of a case study reported in the seminal paper by Miralbell et al. (69), now including dose and risk from stray neutron exposures from passively scattered and scanned proton beams. The results confirmed the qualitative finding of Miralbell et al., namely, that proton therapy conferred lower risk than photon therapy. The modeling study contradicted Hall’s speculation that scanned proton beams provide substantially lower risk compared with scattered proton beams. It also increased knowledge of the mean radiation weighting factor for neutrons, allowing meaningful comparisons of proton and photon CSIs, in spite of the large uncertainty in the neutron RBE for carcinogenesis. Limitations of the study included the use of linear non-threshold risk models for radiation protection (70), the use of a stylized adult phantom, and simplified treatment planning. In rapid succession, these limitations were overcome in a series of papers on CSI that included newer risk models for radiogenic cancers (71), relaxed assumptions about linear non-threshold (LNT) risk behavior (72), personalized voxel phantoms (73), and highly realistic treatment planning methods (32). Another study reported ways to reduce stray radiation from passively scattered CSI to levels approaching those from scanned beams (74). Conversely, adding final collimators to scanned beams may sharpen the lateral penumbra, thereby reducing dose just outside the target (75, 76), which increases the neutron leakage exposure. Findings common to these studies are that many small details matter and that it is difficult to know *a priori* which details will have a profound effect on risk after CSI. An example of a treatment factor of importance is the superior–inferior location of the junction of the abutting cranial and upper spinal fields, which can have a profound effect on thyroid risk. Another is the selection of margin size on the spinal fields, which has a strong impact on the risk to lung and other organs. Details of the research methodology also matter, such as the exclusion of the contents of the bladder and colon when delineating the organs and tissues at risk (77) and blood in the heart (35).

In the last 5 years, much progress has been made toward practical analytical models of neutron leakage exposures. A simple analytical model was proposed in 2010 (78) for 250 MeV beams, improved and extended to cover the 100–250-MeV proton-beam energy interval (67, 79), and validated for low-energy proton beams for ocular treatments (79, 80). The analytical model was extended to include range modulation and implemented in a research TPS (81).

Analytical models of exposures from neutrons generated inside the patient were investigated by Schneider et al. (82), who reported a simple parameterization for a spherical water phantom. Currently, analytical algorithms are lacking for predicting internal neutrons.

#### Photon Therapy

In the early 2000s, intensity modulated photon radiation therapy (IMRT) was widely deployed. Hall and Wuu pointed out that the fluence modulation increases the monitor units by a factor of 2–3, thereby proportionately increasing the whole-body exposures from leakage radiation (83). They estimated this could lead to a doubling of the second cancer incidence at 10 years. Hence, the improved sparing of normal tissue immediately surrounding the tumor comes at the cost of increased exposure of the whole body to leakage radiation.

Generally speaking, commercial TPSs use deterministic dose algorithms that provide adequate accuracy in-field and near-field. However, approximately 10 cm distance or more outside the treatment field, the accuracy is typically worse than 40% and deteriorates dramatically with increasing distance. Because of the high incidence of late effects that are observed outside the treatment field, it will be essential to solve this problem, i.e., to achieve a dosimetric accuracy of 20% or better in all tissues.

Specialized Monte Carlo models were developed in 1980s for external beam radiotherapy research. One widely used code is based on the Electron Gamma Shower (EGS) code (84) with an add-on module (BEAM) (85) to compute photon and electron fluences emanating from an electron linac. Another add-on module named DOSExyz (86) facilitates dose computation in matrices of voxels, e.g., from CT image sets. Several fast Monte Carlo codes have been developed for radiotherapy dosimetry applications, including Voxel Monte Carlo (VMC) (87), dose planning method (DPM) (88), and MCDOSE (89). Most of the fast Monte Carlo calculation codes are designed for in-field or near-field dose calculations. General purpose codes, such as MCNP (39) and GEANT4 (44), include physics models for the production and transport of photoneutrons and have been used in many types of radiotherapy sources and clinical accelerators. At least one commercial TPS offers an electron Monte Carlo algorithm (90, 91). There has been effort to combine the commercial TPS and calibrated fast Monte Carlo codes to provide organ dose calculations in both in-field and out-of-field regions for epidemiological studies (92). The Monte Carlo method has been an invaluable research tool for studying therapeutic and stray radiation exposures.

Analytical models have been used to predict stray radiation exposures for several decades for conventional radiotherapy (30). However, the literature on models for *contemporary* treatment units and *advanced* treatment techniques has been extremely limited, as recently reviewed in Ref. (38). An empirical model was developed for photon CSI (93, 94) to model out-of-field radiation exposures. That model parameterized measured data in an approach conceptually similar to that of Stovall et al. (95). Recently, a fast and simple physics-based analytical model was reported for a widely used type of medical linear accelerator (38). This approach is currently being further validated for use with 12 different types of linacs and treatment techniques, including advanced technology treatments and techniques, such as IMRT, volumetric modulated arc therapy (VMAT), IMRT with flattening-filter-free (FFF) beams, tomotherapy, and robotic-arm linac therapy. The advantages of a physics-based approach include reduced requirements for measured data, increased predictive capabilities, broader applicability, fast computation time, and simplicity compared to Monte Carlo models. Analytical models for advanced technology radiotherapies are progressing rapidly but not yet sufficiently developed for routine use in clinical treatment planning. Major challenges lie in balancing the competing requirements of simplicity, dosimetric accuracy, and applicability. With the increasing diversity of advanced radiotherapies, it is not yet clear if this approach will be able to simultaneously meet all requirements.

Measurements have long been considered the most reliable source of information on stray radiation exposures and are needed to validate predictive models. For advanced technology photon radiotherapies, numerous measurements have been reported in air, water-box phantoms, or anthropomorphic phantoms, such as those in Ref. (38, 64, 96, 97). Most advanced technology photon therapies are administered at photon energies below the threshold for photoneutron production. For this reason and for brevity, exposures from photoneutrons are not discussed further.

#### *In Silico* Clinical Trials

In many situations, it is difficult or impossible to carry out observational studies, including randomized clinical trials and radiation epidemiology studies. We briefly review reasons for this that are relevant to the advanced technology radiotherapies.

There is an inherent challenge to understanding the late effects from “advanced technology” treatments. Specifically, by the time the late effects manifest, the treatment under investigation becomes standard or obsolete. The latency time for solid tumors typically is longer than 5 years and may be several decades. This problem is exacerbated by the decreasing technology lifetimes because of accelerating technological progress. Other potentially challenging factors to conducting clinical and other observational trials include requirements on equipoise, accrual of sufficient patients to achieve statistical power and significance, and high costs.

However, good decision making in the clinical and policy arenas is informed by the best available evidence. If observational evidence is not available, evidence can be generated using the alternative strategy of computer simulated or *in silico* trials. *In silico* trials utilize representative cohorts (including detailed volumetric images of patient anatomy), clinically detailed and realistic treatment planning methods, physically complete dose assessments (therapeutic and stray radiation exposures), dose–response functions (risk models) from previous observational studies, and rigorous uncertainty analyses. Comparative *in silico* studies use paired data (both the standard and the experimental treatments are simulated for each patient). *In silico* studies are faster and less expensive than traditional observational trials, although their ultimate role will be complimentary rather than competitive.

A lack of equipoise is perhaps the greatest barrier to conducting a randomized clinical trial comparing photon versus proton-beam CSI. By the mid 2010s, the remarkable advances in research methodologies made it possible to perform *in silico* clinical trials that compared predicted risks after proton and photon CSI. The dose reconstructions included whole-body calculations of therapeutic and stray radiation, all the major tissues and organs of the bodies, clinical realism, and the largest cohort studied (*n* = 17) with complete dose reconstructions. The quantities reported included organ doses (98), radiogenic cancer risk (35), and cardiac toxicity (35, 99).

The *in silico* approach enabled systematic exploration of the influence of host factors on predicted risk, including age at exposure, attained age (72), anatomic stature, and sex on predicted risk (35, 100). Studies were performed to explore the risks of various cancer endpoints, including incidence, mortality, excess relative risk, excess absolute risk, lifetime attributable risk, and ratios of various risk quantities as well as non-cancer endpoints, such as cardiac toxicity (35), fertility complications (68), and radiation-induced necrosis (101).

Some progress has been made in understanding the uncertainties in comparative risk predictions in the radiotherapy setting, but many important questions remain open. Uncertainties in risks of radiogenic cancers were reviewed in Ref. (102, 103). The role of sensitivity tests to assess the impact of poorly known uncertainties in biologic aspects of risk comparisons after CSI was demonstrated by Newhauser et al. (33). Specifically, they showed that significant results can be obtained despite the large uncertainties in the RBE of neutrons for carcinogenesis. Also for CSI, Zhang et al. (72) reported on the influence of the risk model selected, including deviations from LNT behavior due to effects, such as cell sterilization at therapeutic doses. Uncertainties of *in silico* trials comparing predicted risks of late effects were investigated using a rigorous propagation of errors by Fontenot et al. (104) and subsequently extended by Rechner et al. (77), Zhang et al. (99), and Nguyen et al. (105). These studies all depend to some extent on the assumption that the extrapolation of risks from low-dose acute exposures to high-dose fractionated exposure is the same for all cancer sites. This might vary from one organ or tissue to the next (106), e.g., due to differences in stem cell repopulation in different organs. Additional progress is needed to quantify the uncertainties in risk models used in comparative studies, such as possible organ-specific variations in the transportation of risk models from Japanese survivors of nuclear detonations to other populations.

Given the many uncertainties in the risk modeling, it is essential to take on the challenge of developing epidemiological and clinical studies to assess the late effects of proton therapy directly, particularly in children who are known to be more radiosensitive to some cancers. The first randomized trial to compare the late effects of proton with photon therapy for breast cancer treatment was recently funded by PCORI and includes 22 US proton centers with an aim of randomizing approximately 2000 women.^1^ This study, which was not yet recruiting patients at the time of this writing, will test the hypothesis that proton therapy reduces cardiovascular disease risks compared to photon therapy. The Pediatric Proton Consortium Registry is currently open to enroll children treated with proton radiation in the United States with the goal to characterize the population receiving proton therapy, regardless of technology used, to evaluate its benefits over other radiation therapies (107). Smaller trials are underway to compare effectiveness in prostate, lung, and head and neck cancer. As far as we are aware, no large-scale randomized trials or epidemiological studies of proton therapy in children are currently underway. As noted previously, they are the patients who are at greatest risks for radiation-related second cancers. The American Society for Therapeutic Radiation Oncology advises against using proton therapy in common cancers, such as prostate cancer, outside well-designed clinical trials.^2^ As the number of proton therapy centers continues to expand in the US, Europe, and Asia, concerted efforts should be made to directly study the late effects of this treatment, especially the development of infrastructure to assess whole-body exposures.

### Related Technologies

Several related technologies have had an enabling effect of methodological research. First and foremost is the remarkable advancement in the capabilities and accuracy of general-purpose Monte Carlo codes, a workhorse of research on medical dosimetry. In fact, the field of radiation oncology owes a debt of gratitude to the nuclear physics community for providing general-purpose Monte Carlo simulation codes and outstanding support, all at little or no cost to the medical community. Whole-body simulations, which have appeared in the literature recently (100), were considered computationally intractable at the turn of the century because of limitations of the Monte Carlo codes’ capabilities, as well as computational expense.

Advances in high-performance computing have had a profoundly enabling effect on computational dosimetry. The most important developments have been the proliferation of low-cost, high-reliability, and parallel computing methods. Several approaches have been used, including clusters of CPUs (108), grid technologies (109), and graphics processing units (110).

The second most important enabling technology is the electronic medical record, which allows a high degree of automation and dependability. This includes standardization of file formats, communications protocols, and interoperability. This has been helpful to researchers, for example, in performing post-processing tasks, such as anonymization of electronic radiotherapy medical records (111), and will become vitally important for outcome studies in the future.

**Figure 3:**
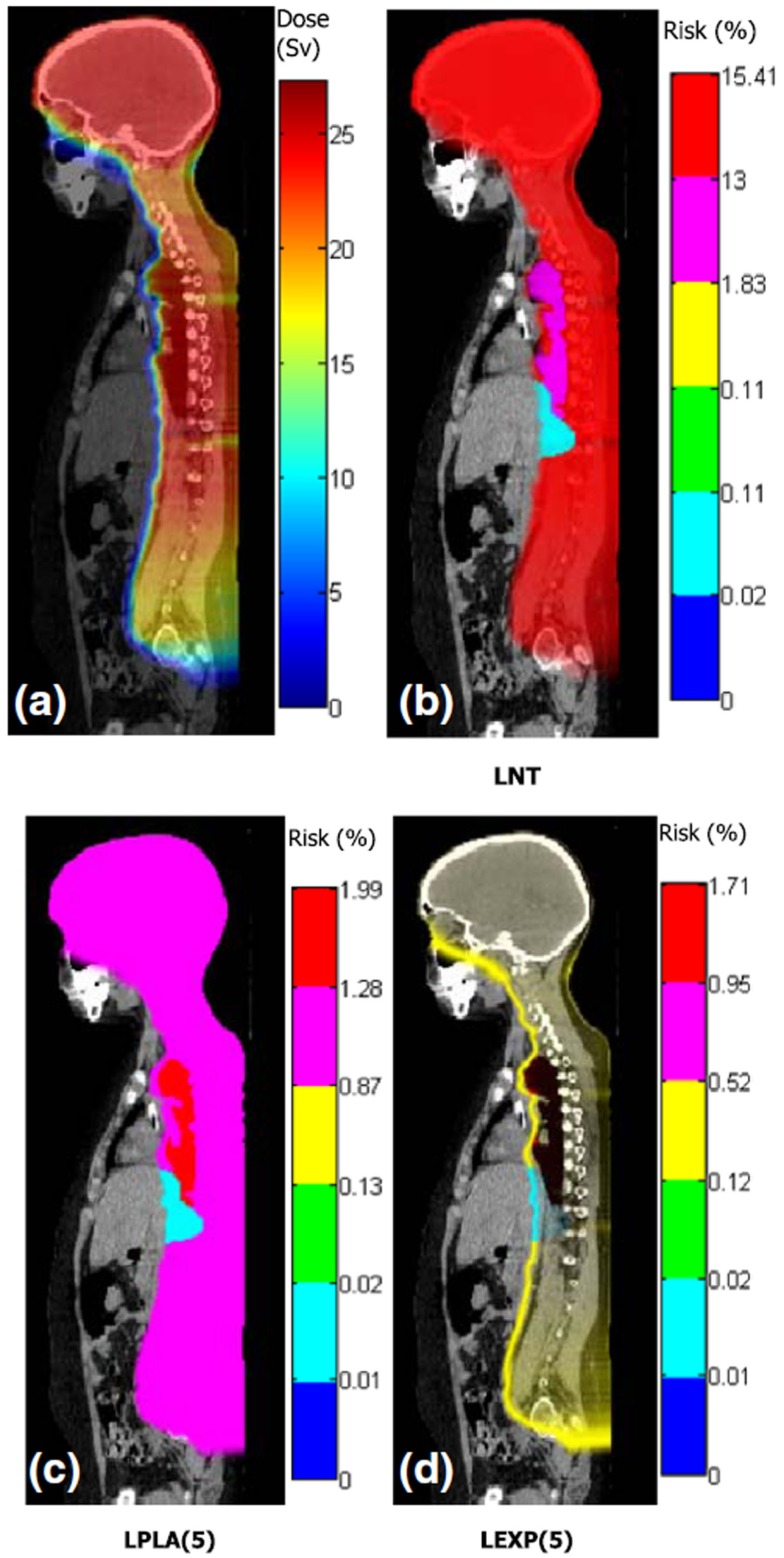
**Distributions of dose and risk superposed on sagittal images of anatomy for craniospinal irradiation**. **(A)** shows equivalent dose and **(B–D)** show lifetime risks of second cancer incidence based on different dose–risk relationships [LNT: linear non-threshold model, LPLA (5): linear plateau model with bending point at 5 Sv, and LEXP (5): linear exponential model with bending point at 5 Sv]. Figure from Ref. (112).

The visualization of radiation risk and detriment will likely become important in the future. In particular, it appears interesting to visualize risks superposed on images of patient anatomy, much like radiation exposure is visualized in contemporary treatment planning (Figure 3). However, for a given dose distribution, the distribution of risk may be radically different due to variations in the radiation sensitivity of individual organs and tissues (26). From a technical standpoint, meaningful visualization of risk presents many challenges, some of which may be overcome (26, 112). Risk visualization methods are nascent and are currently unavailable in contemporary TPSs.

Given the progress in capabilities to predict radiation exposures and risks, it has become technically possible to develop methods to algorithmically minimize predicted risk (113). Such methods could become an automatic step in routine radiotherapy treatment planning. Algorithmic minimization of risk of late effects is nascent and is currently unavailable in TPSs. Substantial additional research, development, and validation will be required before this can be used for human use, e.g., in prospective clinical decision making regarding the care individual patients receive.

## Discussion

Worldwide, the number of incident cancer cases is expected to increase over the next decade. Cancer survival rates are expected to increase further with improved diagnosis, treatment, and survivorship care. For these and other reasons, additional attention must be paid to reduce the incidence of treatment-related morbidity, such as fatal radiogenic second cancers.

Achieving this goal will require new research strategies and methods to supplement and enhance the traditional ones. In general, each new generation of advanced technology enables the delivery of superior (and more complex) therapeutic and stray dose distributions in the body. The exposures vary with a wide variety of treatment factors and host factors. To prospectively assess the full potential of advanced technology treatments to improve outcomes, new methods and capabilities are urgently needed to assess radiation exposures. Promising recent studies, including several examples mentioned in this review article, suggest that it might become feasible to routinely predict radiation exposures to all the tissues and organs of the body in the near future.

At the present time, clinical TPSs provide acceptable dosimetric accuracy for exposures to therapeutic radiation. However, outside the treatment field, the accuracy is generally poor and at distant locations, the exposures are typically not calculated at all. Additional research and development will be needed to develop dose prediction algorithms that provide an acceptable compromise of dosimetric accuracy, computational speed, and ease of use.

To accomplish that, we will need to know much more about the processes governing radiation exposures and risks. In recent years, our understanding of the magnitude and systematics of stray radiation exposures has increased dramatically for advanced proton and photon therapies and further progress is anticipated. However, many key uncertainties remain regarding the magnitude of risks from high-dose fractionated exposures, critical substructures of organs (such as the heart), applicability of risk models based on data from the Japanese atomic bomb survivors to non-Japanese populations, and the RBE of neutrons. From the literature, a coherent picture is emerging in which stray exposures are generally numerically small but the corresponding risks may be large, e.g., 30% or larger lifetime risk of second cancer for some patients. Risks of radiation late effects are of particular concern for patients of young age and with good prognoses for long-term survival.

Advances in research methodologies and capabilities will be necessary. Some key needs for research and clinical settings include

(1) Algorithms to calculate exposures to tissues outside the treatment field.
(2) Primary and secondary reference fields of high-energy neutrons to allow for calibration of instruments to measure exposure.
(3) Improved methods to reproducibly and consistently determine radiation quality.
(4) Methods to accumulate exposures from therapeutic and imaging procedures.
(5) Tools for risk visualization, analysis, communication, and documentation.
(6) Algorithmic methods to support decision making in avoiding non-essential radiation risks.

Ideally, these will be implemented in ways that support the critical need for efficient, large-scale studies of the radiation late effects of proton therapy. When implemented, this will facilitate multidisciplinary research that integrates key aspects of radiation oncology (114), epidemiology (115, 116), physics (117), and survivorship (118, 119). In addition, this may be relevant to some radiobiologic research, such as abscopal (120) and other non-targeted effects (e.g., radiation-induced bystander effect, genomic instability, and the radiation response of stem cells) (121) and modulation of radiation response (e.g., radiosensitizers and radioprotectors) and novel combination therapies (122). The American Association of Physicists in Medicine pointed out potential dangers of using biological models for clinical radiotherapy and provided guidelines and methodology for quality assurance (123).

## Conclusion

In past decades, studies of medical exposures have increased knowledge of radiation risks. However, dose–response functions have large uncertainties because many studies lacked the large sample sizes and high-quality radiation exposure data needed to more accurately estimate risk. Now, for the first time, it appears within reach, scientifically and technically, to prospectively calculate complete, whole-body exposures to virtually all major advanced technology radiotherapy patients. This will be possible because of widespread adoption of the electronic medical record, improved understanding of the physics of stray radiation exposures, advances in high-performance computing, and advances in algorithms to predict radiation exposures. When realized, the availability of exposure data for large populations will open new frontiers of research in radiation epidemiology, clinical oncology, and cancer survivorship.

## Author Contributions

WN drafted the manuscript. AG, CL, and RS edited and expanded the text of the manuscript. All authors reviewed and approved the manuscript.

## Conflict of Interest Statement

The authors declare that the research was conducted in the absence of any commercial or financial relationships that could be construed as a potential conflict of interest.

## Footnotes

^1^http://www.pcori.org

^2^http://www.choosingwisely.org
